# Outcomes following hematopoietic stem cell transplantation in patients treated with standard chemotherapy with or without gemtuzumab ozogamicin for acute myeloid leukemia

**DOI:** 10.1038/s41409-020-01207-4

**Published:** 2021-02-09

**Authors:** Cécile Pautas, Emmanuel Raffoux, Juliette Lambert, Ollivier Legrand, Sylvain Chantepie, Lauris Gastaud, Jean-Pierre Marolleau, Xavier Thomas, Pascal Turlure, Rebecca J. Benner, Erik Vandendries, Karïn Gogat, Hervé Dombret, Sylvie Castaigne

**Affiliations:** 1 Service d’Hématologie et de thérapie cellulaire, Hôpital Henri Mondor, Créteil, France; 2 Hôpital Saint-Louis (AP-HP), EA 3518, Université de Paris, Paris, France; 3 Service d’Hématologie et Oncologie, Centre Hospitalier de Versailles, Le Chesnay, France; 4 Service d’hématologie clinique et de thérapie cellulaire, Hôpital Saint-Antoine (AP-HP), Université Paris Pierre et Marie Curie, Paris, France; 5 Institut d’Hématologie de Basse-Normandie, Caen, France; 6 Service hématologie, Centre Lacassagne, Nice, France; 7 Service hématologie, CHU Amiens, Amiens, France; 8 Service hématologie, Centre Hospitalier Lyon Sud, Lyon, France; 9 Service hématologie et de thérapie cellulaire, CHU de Limoges, Limoges, France; 10 Global Product Development, Pfizer Inc, Groton, CT USA; 11 Pfizer Inc, Cambridge, MA USA; 12 Global Clinical Development, Pfizer Inc, Paris, France; 13 Service d’Hématologie et Oncologie, Centre Hospitalier de Versailles, Université de Versailles Saint Quentin, Le Chesnay, France

**Keywords:** Acute myeloid leukaemia, Acute myeloid leukaemia

## Abstract

The phase 3 ALFA-0701 trial demonstrated improved outcomes with fractionated-dose gemtuzumab ozogamicin (GO) combined with standard chemotherapy vs. standard chemotherapy alone in adults with de novo acute myeloid leukemia (AML). We examined post-transplant outcomes and occurrence of hepatic veno-occlusive disease/sinusoidal obstruction syndrome (VOD/SOS) in patients who received hematopoietic stem cell transplantation (HSCT) as follow-up therapy in ALFA-0701. Patients aged 50–70 years were randomized to standard chemotherapy with or without GO (3 mg/m2 on days 1, 4, and 7 of induction and day 1 on each of two consolidation courses). Allogeneic HSCT was recommended for patients in first complete remission with matched (related or unrelated) donor, except those with core-binding factor AML or normal karyotype and either NPM1+/FLT3-ITDwt or CEBPA+ AML. Eighty-five patients (GO: *n* = 32; control: *n* = 53) received HSCT in first complete remission or after relapse/primary induction failure. Three patients (GO: *n* = 2; control: *n* = 1 [received GO as follow-up therapy]) developed VOD/SOS after HSCT or conditioning. Post-transplant survival, non-relapse mortality, and relapse were not different between arms. Results indicate fractionated-dose GO as part of induction and consolidation chemotherapy for AML does not induce excess post-transplant VOD/SOS or mortality and thus does not preclude the use of HSCT as consolidation treatment.

Gemtuzumab ozogamicin (GO), a humanized anti-CD33 monoclonal antibody linked to calicheamicin, is indicated for the treatment of adult and pediatric (aged ≥ 1 month) patients with newly diagnosed CD33-positive (CD33+) acute myeloid leukemia (AML) and adult and pediatric (aged ≥ 2 years) patients with relapsed/refractory CD33+AML [1]. The phase 3 ALFA-0701 trial demonstrated significant improvement of event-free and relapse-free survival with fractionated-dose GO added to standard chemotherapy vs. standard chemotherapy alone [2, 3]. Veno-occlusive disease/sinusoidal obstruction syndrome (VOD/SOS) is a concern with GO, particularly when administered before hematopoietic stem cell transplantation (HSCT) [4]. However, lower, fractionated GO dosing may mitigate this risk [5, 6].

This retrospective analysis examined VOD/SOS and post-transplant outcomes in patients who received HSCT as follow-up therapy in ALFA-0701. Study design and patient eligibility have been described [3]. Briefly, 271 patients, aged 50–70 years, with previously untreated de novo AML were randomized to a 3 + 7 induction course of daunorubicin (days 1–3) and cytarabine (days 1–7) with or without GO 3 mg/m^2^ (maximum dose 5 mg; days 1, 4, and 7). Patients in complete remission (CR) following induction received two consolidation courses of daunorubicin and cytarabine with or without GO (3 mg/m^2^ on day 1) according to their initial randomization. Patients with delayed count recovery were not given GO for consolidation. Allogeneic HSCT was recommended for patients in first CR with matched (related or unrelated) donor, except patients with core-binding factor AML or with normal karyotype and either *NPM1*+/*FLT3-*ITDwt or *CEBPA*+AML. A 2-month interval between last GO dose and HSCT was recommended. Conditioning type was left to the discretion of the transplant center.

Data on VOD/SOS were collected as described [2]. Diagnosis and grading were based on investigator judgment. VOD/SOS was classified as pre- or post-transplant relative to first HSCT date. Time-to-event endpoints were post-transplant survival, non-relapse mortality (NRM), relapse, and overall survival (OS; see Supplementary Methods).

ALFA-0701 was approved by the ethics committee of Saint-Germain en Laye, France, and institutional review board of the French Regulatory Agency, and conducted in compliance with the Declaration of Helsinki. All patients provided written informed consent (EudraCT Number, 2007-002933-36; ClinicalTrials.gov, NCT00927498).

Eighty-five patients (GO: *n* = 32; control: *n* = 53) received HSCT as follow-up therapy. One patient received autologous HSCT. Eight patients in the control arm received GO as follow-up therapy before HSCT, and one patient in the GO arm received HSCT but not GO, totaling 39 patients (GO: *n* = 31; control: *n* = 8) who received GO before HSCT.

Baseline and transplant characteristics were generally similar between arms (Table S1). In the GO and controls arms, respectively, transplant was performed during first CR in 53.1 and 41.5%, after induction failure in 6.3 and 17.0%, and after relapse in 40.6 and 41.5% of patients. One patient received HSCT<2 months after last GO dose (as follow-up therapy). Most patients received reduced-intensity conditioning (GO: 78.1%; control: 75.5%). In patients who achieved CR in the GO (*n* = 29) and control (*n* = 44) arms, respectively, median time from first CR to HSCT was 6.4 and 7.8 months.

Post-transplant/conditioning VOD/SOS occurred in three patients (GO: *n* = 2; control: *n* = 1; Table S2), for a rate of 6.5% (*n* = 2/31) among GO-treated patients in the GO arm and 7.7% (*n* = 3/39) among all patients receiving GO before HSCT. Of these, one patient in the GO arm first developed VOD/SOS during induction. GO was permanently discontinued. The patient recovered within ~1.5 months and subsequently received two allogeneic HSCTs with reduced-intensity conditioning. VOD/SOS occurred again after second HSCT from an HLA-matched donor (relatedness unknown) following rejection of first HSCT; symptoms included painful hepatomegaly and moderate cholestatic jaundice. No treatment was given. The patient fully recovered within ~1 month but died ~14.8 months after first HSCT.

The second patient in the GO arm developed VOD/SOS after reduced-intensity conditioning but before allogeneic HSCT from an HLA-matched unrelated donor. Symptoms included weight gain, abdominal distension with frank ascites, peritoneal effusion revealed by ultrasound, and increased liver function tests with cytolysis. Severe sepsis was a concomitant event. Treatment included defibrotide. After progressive normalization of liver function tests within 10 days, the patient experienced a recurrence of cholestatic injury with hepatocellular injury associated with respiratory distress, leading to the patient’s death within a month of HSCT. VOD/SOS was not resolved at the time of death.

The third patient, who was in the control arm and received GO as follow-up therapy before HSCT, developed VOD/SOS after autologous HSCT with myeloablative conditioning. Symptoms included hepatomegaly with signs of portal hypertension and intraperitoneal effusion with thickening of gallbladder walls revealed by abdomen ultrasound, and increased total bilirubin and liver enzymes. Treatment included furosemide and heparin. The patient fully recovered within 1 month and was still alive ~28.7 months after HSCT.

VOD/SOS occurred before HSCT in three patients (GO: *n* = 2; control: *n* = 1; Table S3). Both patients in the GO arm developed VOD/SOS during induction and fully recovered. One of these patients developed VOD/SOS again after second HSCT (described above). The patient in the control arm developed VOD/SOS after follow-up therapy with GO and cytarabine and fully recovered.

Overall, post-transplant outcomes did not differ between treatment arms (Fig. 1). In the GO vs. control arm, median survival was 21.4 vs. 17.1 months; the 12-month rate (95% confidence interval [CI]) of NRM was 28.7% (14.1–45.2) vs. 21.6% (11.5–33.8) and relapse 9.4% (2.3–22.6) vs. 31.4% (19.1–44.4). Post-transplant survival did not differ between arms in patients receiving HSCT in first CR or after relapse/induction failure (Fig. S1). OS did not differ between arms in patients who received HSCT (hazard ratio [HR] 0.97; 95% CI: 0.54–1.75; *P* = 0.9190; median OS: 34.0 vs. 32.5 months), but was longer in the GO vs. control arm in patients who did not receive HSCT (HR 0.68; 95% CI: 0.48–0.97; *P* = 0.0333; median OS: 23.7 vs. 14.9 months).

**Fig. 1:**
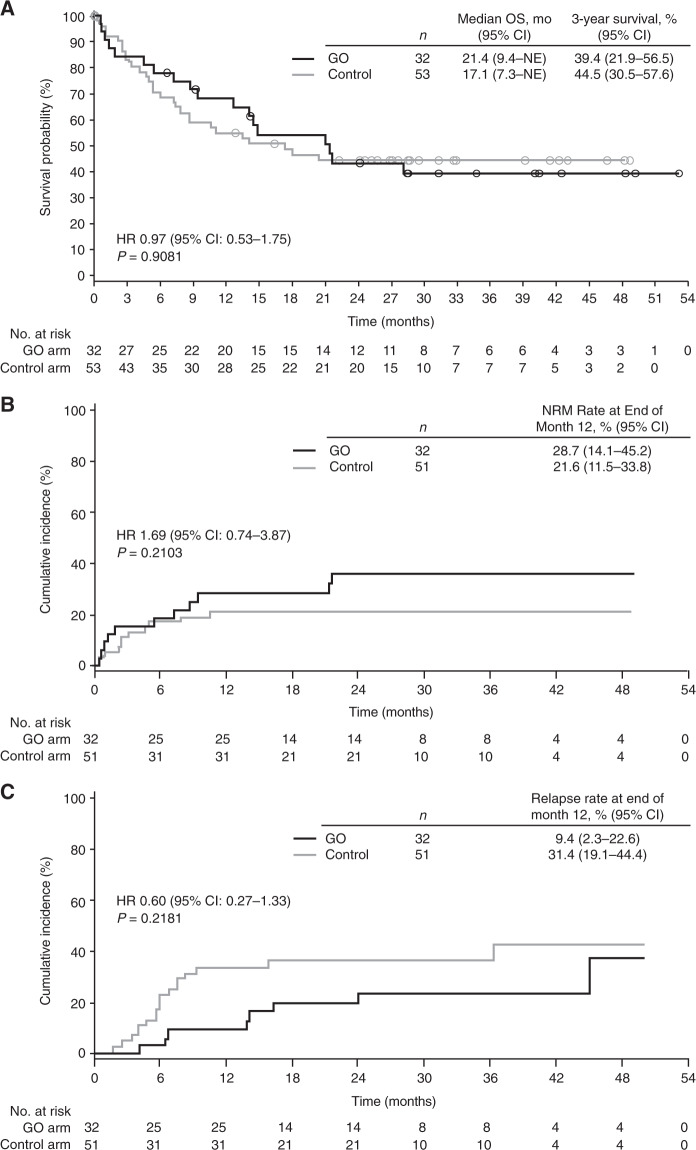
Post-transplant outcomes. Post-transplant outcomes in all patients who received HSCT including **A** post-transplant survival, **B** cumulative incidence of NRM, and **C** cumulative incidence of relapse. CI confidence interval, GO gemtuzumab ozogamicin, HR hazard ratio, HSCT hematopoietic stem cell transplantation, NE not estimable, NRM non-relapse mortality, OS overall survival.

Our findings indicated that fractionated-dose GO added to standard chemotherapy was associated with a low incidence of VOD/SOS after HSCT in this cohort. The rate of post-transplant/conditioning VOD/SOS in GO-exposed patients (7.7%) was consistent with other recent retrospective reports of GO-exposed patients who received HSCT [7, 8]. Fractionated dosing, the use of reduced-intensity conditioning in most patients, and the 2-month interval between last GO dose and HSCT, followed in all but one patient, may have contributed to our low observed rate. Another small study of patients with high-risk, relapsed/refractory AML who received fractionated-dose GO plus chemotherapy found that no patient who underwent allogeneic HSCT—the majority with reduced-intensity conditioning—developed VOD/SOS [6]. It should be noted that VOD/SOS prophylaxis data were not systematically collected in our study; therefore, we cannot exclude the possibility that some patients may have received prophylactic defibrotide.

Post-transplant outcomes were similar between arms, although the small sample size may have precluded the detection of small differences in outcomes. Alternatively, transplant may have eliminated any differences in outcomes between arms, as OS was improved in the GO vs. control arm in patients who did not receive HSCT, but did not differ between arms in patients who received HSCT.

In conclusion, these findings suggest that fractionated-dose GO as part of induction and consolidation chemotherapy for adult AML does not induce excess post-transplant mortality and VOD/SOS and thus does not preclude the use of HSCT as consolidation treatment following induction or salvage treatment.

## Supplementary information

Supplemental Material
