# Supplementary material for: Outcomes following hematopoietic stem cell transplantation in patients treated with standard chemotherapy with or without gemtuzumab ozogamicin for acute myeloid leukemia
Source: Bone Marrow Transplant. 2021 Feb 9;56(6):1474–7. doi: 10.1038/s41409-020-01207-4 (PMC8189917; doi:10.1038/s41409-020-01207-4)
Supplement: Supplementary file 1 — Supplemental Material [file 41409_2020_1207_MOESM1_ESM.pdf]

Supplementary Methods

Statistical Analyses

Time-to-event outcomes:

- Post-transplant survival: time from first HSCT to death due to any cause;
- Non-relapse mortality (NRM): time from first HSCT to death due to any cause without prior relapse;
- Relapse: time from first HSCT to relapse or death related to relapse;
- Overall survival (OS): time from randomization to death due to any cause.

Probabilities of post-transplant survival and OS were estimated using the Kaplan–Meier method, and arms were compared by the log rank test (two-sided *P*-values presented). Hazard ratios (HRs) were calculated using the Cox proportional hazards model. Cumulative incidence rates of NRM and relapse were estimated adjusted for the competing risk of relapse or relapse-related death for the NRM analysis, and death unrelated to relapse for the relapse analysis. Subdistribution HRs were calculated based on Fine and Gray [1], and arms were compared by Gray's test (two-sided *P*-values presented). The data cutoff date for these outcome measures was 30 April 2013.

Reference

1. Fine JP, Gray RJ. A proportional hazards model for the subdistribution of a competing risk. *J Am Stat Assoc.* 1999; **94:** 496–509.

Table S1. Baseline and transplant characteristics

n (%)[a]	**GO arm** *n* **= 32**	**Control arm** *n* **= 53**
**Baseline characteristics**		
Age, median (range), years	60 (51–67)	59 (50–69)
Male	15 (46.9)	24 (45.3)
ECOG performance status		
0	13 (40.6)	20 (37.7)
1	17 (53.1)	29 (54.7)
2	2 (6.3)	4 (7.5)
European LeukemiaNet risk[b]		
Favorable/intermediate	20 (62.5)	38 (71.7)
Poor/adverse	9 (28.1)	13 (24.5)
Unknown	3 (9.4)	2 (3.8)
Cytogenetics[c]		
Favorable	0	1 (1.9)
Intermediate	25 (78.1)	37 (69.8)
Unfavorable	4 (12.5)	11 (20.8)
Unknown	3 (9.4)	4 (7.5)
**Transplant characteristics**[d]		
Time from last GO dose to transplant, median (range), days[e]	222 (64–1215)	121 (52–753)
Timing relative to last GO dose[f]		
<2 months	0	1 (1.9)
≥2 months	31 (96.9)	7 (13.2)
Timing of transplant relative to EFS event[g]		
In first complete remission	17 (53.1)	22 (41.5)
After relapse	13 (40.6)	22 (41.5)
After induction failure	2 (6.3)	9 (17.0)
Disease status at time of transplant		
In complete remission	27 (84.4)	46 (86.8)
Not in complete remission	3 (9.4)	6 (11.3)
Transplant type		
Allogeneic	32 (100)	52 (98.1)
Autologous	0	1 (1.9)

n (%)[a]	**GO arm** *n* = 32	**Control arm** *n* = 53
Donor relatedness		
Related	10 (31.3)	23 (43.4)
Unrelated	20 (62.5)	27 (50.9)
Unknown	2 (6.3)	3 (5.7)
HLA compatibility		
Matched	27 (84.4)	44 (83.0)
Unmatched	2 (6.3)	4 (7.5)
Unknown	3 (9.4)	5 (9.4)
Donor relatedness/HLA compatibility		
Matched/related	9 (28.1)	23 (43.4)
Alternative donor	19 (59.4)	24 (45.3)
Unknown	4 (12.5)	6 (11.3)
Stem cell source		
Bone marrow	7 (21.9)	18 (34.0)
Peripheral blood	20 (62.5)	29 (54.7)
Cord blood	4 (12.5)	4 (7.5)
Unknown	1 (3.1)	2 (3.8)
Conditioning type		
Myeloablative	5 (15.6)	9 (17.0)
Reduced intensity	25 (78.1)	40 (75.5)
Unknown	2 (6.3)	4 (7.5)
Conditioning regimen included busulfan + fludarabine	14 (43.8)	29 (54.7)
Conditioning regimen included busulfan + cyclophosphamide	2 (6.3)	6 (11.3)

[a]Unless otherwise noted

[b]Risk status based on 2010 guidelines [2]

[c]Cytogenetic classification was defined as follows:
Favorable included inv(16)/t(16;16) and t(8;21);
Unfavorable included monosomy 5 or del(5q), monosomy 7 or del(7q), t(6;9), t(9;22), 3q26 abnormalities except t(3;5), 11q23 abnormalities except t(9;11), and complex karyotypes with ≥3 abnormalities;
Intermediate included all other anomalies as well as normal karyotypes (karyotype classified as normal when ≥20 mitoses without chromosomal anomalies were observed in bone marrow specimens)

[d]Data based on first HSCT and conditioning regimen

[e]Eight patients in the control arm received GO as follow-up therapy prior to HSCT

[f]One patient in the GO arm received HSCT but not GO

[g]Patients who received transplant after relapse or induction failure could have achieved subsequent complete remission prior to transplant

ECOG Eastern Cooperative Oncology Group, *EFS* event-free survival, *GO* gemtuzumab ozogamicin, *HLA* human leukocyte antigen, *HSCT* hematopoietic stem cell transplantation

Reference

2. Dohner H, Estey EH, Amadori S, Appelbaum FR, Buchner T, Burnett AK *et al*. Diagnosis and management of acute myeloid leukemia in adults: recommendations from an international expert panel, on behalf of the European LeukemiaNet. *Blood*. 2010; **115:** 453–474.

Table S2. Summary of VOD/SOS events after HSCT or conditioning

	Treatment arm	Timing of VOD/SOS	VOD/ SOS grade	Outcome	Transplant type	Timing of HSCT / Disease status at HSCT	Stem cell source	Conditioning intensity / Regimen	Time between last GO dose and VOD/SOS	Time between HSCT and VOD/SOS
1	GO[a]	After 2nd HSCT	3	Recovered	Allogeneic	In CR1 / In CR1	Cord blood	Reduced intensity / Cyclophosphamide	9 months[b]	A few days
2	GO	After conditioning	4	Died before VOD/SOS resolved	Allogeneic	After relapse / In later CR	Peripheral blood	Reduced intensity / Fludarabine and busulfan	301 days	N/A
3	Control[c]	After HSCT	3	Recovered	Autologous	After relapse / In later CR	Peripheral blood	Myeloablative / Busulfan and cyclophosphamide	75 days	25 days

[a]Patient had two VOD/SOS events, one prior to first HSCT and one after second HSCT. Second HSCT and second VOD/SOS event described here

[b]After last dose of chemotherapy. GO was discontinued following induction due to first occurrence of VOD/SOS

[c]Patient received GO as follow-up therapy in combination with cytarabine after relapse

CR complete remission, *GO* gemtuzumab ozogamicin, *HSCT* hematopoietic stem cell transplantation, *VOD/SOS* veno-occlusive disease/sinusoidal obstruction syndrome

Table S3. Summary of VOD/SOS events before HSCT

	Treatment arm	Grade	Outcome	Time between last GO dose and VOD/SOS
1	GO[a]	3	Recovered	10 days
2	Control[b]	4	Recovered[c]	49 days
3	GO	2	Recovered[d]	~28 days

[a]Patient had two VOD/SOS events, one prior to HSCT and one after second HSCT. GO was permanently discontinued after the first event, described here

[b]Patient received GO as follow-up therapy

[c]Patient received HSCT on 13 June 2013 and was still alive as of the 1 November 2013 retrospective data collection cutoff date

[d]Patient was still alive ~48.3 months after HSCT

GO gemtuzumab ozogamicin, *HSCT* hematopoietic stem cell transplantation, *VOD/SOS* veno-occlusive disease/sinusoidal obstruction syndrom

Fig. S1 Post-transplant survival in patients receiving transplant (A) in first complete remission or (B) after relapse/primary induction failure.

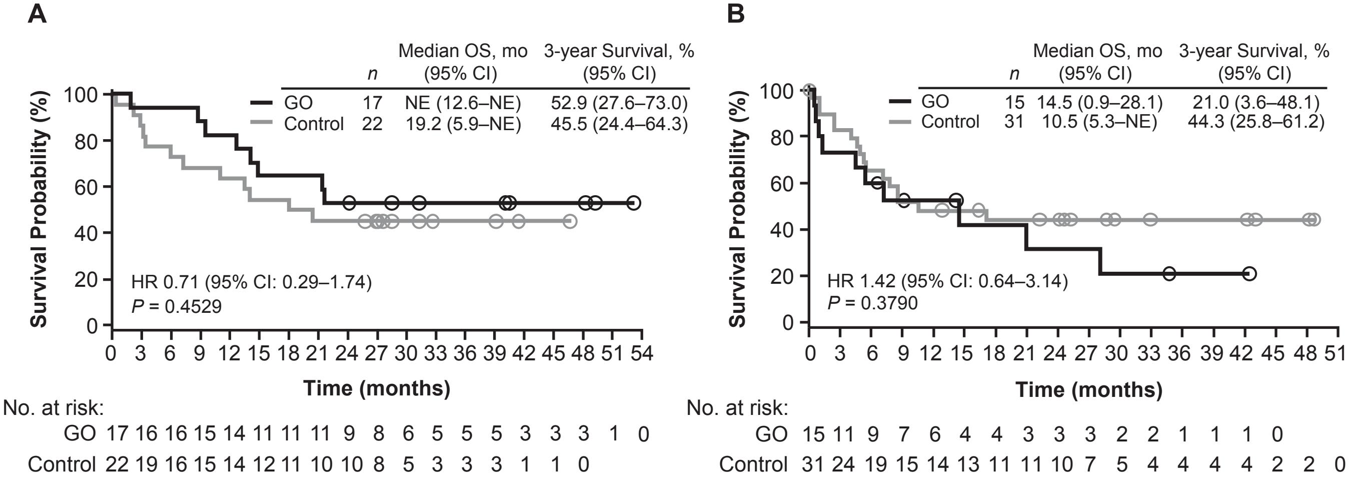

CI confidence interval, *GO* gemtuzumab ozogamicin, *HR* hazard ratio, *NE* not estimable, *OS* overall survival
